# Cervical cancer screening adoption behaviours among Nigerian women in academics: using a health belief model

**DOI:** 10.1186/s12905-024-03380-w

**Published:** 2024-09-27

**Authors:** Adaobi C. Iluno, Frederick O. Oshiname, Adeyemi O. Adekunle, Justin Dansou

**Affiliations:** 1 Faculty of Health Studies, University of Bradford, Bradford, UK; 2 Department of Public Health, Babcock University, Illishan Remo, Ogun Nigeria; 3 University College Hospital, Ibadan, Nigeria; 4 Planning, and Demography (ENSPD), National School of Statistic, University of Parakou, Parakou, Republic of Benin

**Keywords:** Health belief model, Cervical cancer, Cervical cancer screening, Nigerian women in academics

## Abstract

**Background:**

Cervical Cancer is the commonest and one of the leading causes of death from cancer among women in developing countries. Screening has been shown to reduce morbidity and mortality from the illness, yet its uptake is low. This study investigated the pattern of utilization and preferences relating to the adoption of cervical screening among female postgraduate students at the University of Ibadan.

**Methodology:**

The study was a descriptive cross-sectional survey involving the use of a multi-stage sampling technique to recruit 372 women undergoing postgraduate studies (20–52 years with a mean age of 27.3 ± 5.4) at the University of Ibadan, Nigeria. A pretested semi-structured, self-administered questionnaire was used for data collection and the coded data were analyzed using SPSS (version 20).

**Results:**

Only 4.0% of the respondents had been screened for Cervical Cancer at the time of study while 86.3% expressed their desire to be screened if given the opportunity. Most participants showed a favourable perception with 70.4% disagreeing that cervical cancer is a mild disease and 50.5% agreeing that the benefits of cervical cancer screening outweigh the stress of the screening procedure. Female doctors (73.2%) topped the list of health professionals’ respondents who preferred to conduct the screening. A majority (70.7%) of the respondents preferred these screenings to be done during antenatal clinic visits. There is a significant association (*p*.value = 0.0007) between cervical cancer screening behaviors and sexual activity among women.

**Conclusion:**

Poor utilization of Cervical cancer screening services is seen among Nigerian women undergoing postgraduate studies but a high willingness to utilize the services in the future with consideration to professionals delivering the service and specific locations where it can be obtained. The poor rate of cervical cancer screening from the study depicts the large extent to which cases of this cancer go without being detected till the advanced stages. Rolling out more screening strategies that will explore different service delivery points/preferences as highlighted in the study is needed for larger coverage.

## Introduction

Over the past decades, Cervical Cancer (CC) has continued to be a major public health problem affecting women of reproductive age globally. In a global analysis conducted in 2018, it was reported that approximately 570,000 women developed CC and 311,000 women died from it [1]. The study further mentioned the disease as the fourth most common cancer among women, after breast cancer. The global cancer statistics estimated that CC has the highest cancer incidence rate among women in 28 countries and is the most common type of cancer-related mortality among women in 42 countries, the majority being in sub-Saharan Africa [2]. The statistics indicated that while cervical cancer incidence rates are declining in high-resource areas, incidence, prevalence, and mortality rates continue to rise in low and middle-income countries [2]. WHO projected that CC mortality is expected to increase by 42% to 442,926 deaths in the year 2030 [3]. Countries with low resources will have the greatest rise of about 85% incident rate of the disease with 87% deaths occurrence [4].

There are about 50.33 million women of reproductive age in Nigeria who are at risk of developing cervical cancer [2]. One of the most common Sexually Transmitted Infections (STIs) is the Human papillomavirus (HPV) [5]. HPV 16 /18 contributes to 66.9% of invasive CC and about 3.5% of women of reproductive age are estimated to harbour cervical HPV-16/18 infection at a given time [2, 6]. According to the Information Centre’s recent report on HPV, the current estimate indicates that every year 14,943 women are diagnosed with CC in Nigeria, and about 10,403 die from the disease [2].

As the public health burden of cervical cancer increases in Nigeria, there is a need for more strategic efforts towards the vaccination of adolescent girls with HPV vaccines before the onset of sexual activity. Nigeria developed a five-year National Strategic Plan which proposed to vaccinate 78% of girls with the HPV vaccine and screen 80% of women primarily through HPV DNA testing by 2021 [7]. Unfortunately, these ambiguous targets are yet to be achieved due to limited resources available for scale-up.

Cancer Research UK asserts that the detection of CC in its earliest stages is lifesaving and it has been estimated that cervical screening saves around 5,000 lives each year in the UK [8]. Cervical screening can prevent at least, 45% of cervical cancers in women aged 30–39 years, 60% of cervical cancers in women aged 40–49 years, and 75% of cervical cancers in women aged 50–69 [8]. Despite the advantages of screening, screening practice is still generally poor, especially in developing countries. Most women with CC, especially in developing nations, never got screened for the disease before they became symptomatic of it thereby reducing their chances of survival [9]. Interventions have been rolled out in Nigeria to promote cervical cancer screening services such as simple and more comfortable procedures, greater awareness, and even free screening services by some organizations. Yet the coverage of CCS in Nigeria is still poor.

Studies have been conducted to investigate the several barriers that influence the uptake of cervical cancer screening (CCS) such as its financial implications, accessibility, availability, and poor knowledge among women of reproductive age [10–12]. However, few studies have reported women’s preferences toward the delivery of cervical cancer screening. Furthermore, it is presumed that educated women are more informed on health issues and make greater use of health services [13]. The extent to which this is true among Nigerian women in postgraduate studies is yet to be explored. Unfortunately, the current screening practices, future intention to screen, and service preferences among Nigerian women in academics have not been adequately investigated thus limiting the opportunity to plan appropriate educational and service interventions targeted at them. There is an urgent need for more research on this topical issue, especially those with the potential to yield evidence-based information regarding CCS adoption behaviors among female postgraduate students. Such information is essential for designing interventions that would enhance the uptake of CCS services. This study aims to fill these gaps in the existing knowledge. It is these gaps in knowledge that necessitated the design of this study.

## Methodology

The study was carried out at the University of Ibadan. The University Health Centre also referred to as Jaja Clinic, provides relevant health services such as minor treatments and referrals to the University College Hospital (UCH), Ibadan when necessary. CCS services are not being provided within the Jaja clinic yet, though opportunistic programs have been carried out within the clinic. However, UCH provides cervical cancer screening services at a considerable cost.

The descriptive cross-sectional study design was used to conduct the study among women undergoing postgraduate study and living within the University residential hall at the time of the study. The total population of women living within the University residential hall was 622 and the estimated sample size was calculated as 338. A 10% non-response rate was calculated as 34 and added to the estimated sample size to give a calculated sample size of 372. A multi-stage sampling technique was used to select the 372 study respondents. The quantitative data were collected using semi-structured self-administered questionnaires. The administration of the questionnaires was done in the hall of residence. A participant was interviewed in each of the selected rooms. Informed consent was obtained from the participants before administering the questionnaires. The questionnaires were collected immediately and cross-checked for completeness. Data collection was carried out for three weeks with the assistance of four field officers.

### Data management and analysis

The questionnaires were checked for completeness and a serial number was given to each for easy identification and recall. The responses in each questionnaire were hand-coded with the use of a coding guide. A template was designed on the SPSS (version 18) for entering and analysis of the coded data.

## Results

The result highlighted below using the Health Belief Model (HBM) proposed by Rosenstock (1966) [14], explained, and predicted the likely behavior of African women towards the uptake of cervical cancer screening services.

### Socio-demographic characteristics of respondents

There were more Masters’ students (87.9%) represented than the rest such as Ph.D. (8.9%), MPhil/Ph.D (1.9%), etc. The age range was between 20–52 years with a mean age of 27.3 ± 5.4. The majority (80.3%) were young adults aged 20–29 years, others who were between 30–39 years and 40–49 years constituted 13.9% and 5.1%, respectively. Most (89.8%) were single while 9.9% were married.

### Sexual and reproductive health experiences and vulnerability among respondents

When asked the question “ever had sex” almost half (49.5%) of the respondents stated ‘Yes’ while 50.5% said ‘No’. The age at sexual debut ranged from 8–33 years with a mean of 21.6 ± 4 years. A majority (73.3%) had sex first when they were within the age range of 15–24. 13.5% said they could not disclose their age at sexual debut. Only 9.4% of respondents had ever taken oral contraceptives while 1.3% had a family member who had CC. More details are shown in Table 1.

**Table 1:** Respondents’ sexual and reproductive health experiences

Reproductive and Sexual experiences	No	%
*Ever had sex (N* = *372)*		
Yes	184	49.5
No	188	50.5
*Age at sexual debut*^a^* (n* = *105)*		
5–14	6	5.7
15–24	77	73.3
25–34	22	21
*Other responses (n* = *267)*		
Never had sex/NA	188	70.4
I cannot remember	12	4.5
I cannot say/disclose	66	24.7
Raped	1	0.4
*Ever taken oral contraceptives n* = *371*		
Yes	35	9.4
No	336	90.6
*Family member with a history of cervical cancer N* = *372*		
Yes	5	1.3
No	367	98.7
*Relationship with this family member with a history of cervical cancer n* = *5*		
Cousin	2	40
Mother’s sister	1	20
Father’s sister	1	20
Mother’s cousin	1	20

^a^Mean age at sexual debut = 21.6 ± 4, Median = 22, Mode = 20, Range = 8–33

### Perception towards cervical cancer and cervical cancer screening

Most (70.4%) disagreed that CC is a mild disease while 35.8% were undecided about CC leading to infertility. 12.4% agreed that cervical cancer occurs in sexually active people. See Table 2a for more details.

**Table 2:** (a-b): Respondents’ perception relating to cervical cancer and cervical cancer screening

**a) Perception towards Cervical Cancer**	Agree	Disagree	Undecided
Cervical cancer only occurs in people who are too sexually active	46 (12.4)	165 (44.4)^a^	161 (43.3)
Cervical cancer is a mild disease	12 (3.2)	262 (70.4)^a^	98 (26.3)
Cervical cancer screening can lead to infertility	101 (27.2)	138 (37.1)^a^	133 (35.8)
Cervical cancer occurs only in people who do not know God	6 (1.6)	273 (79.8)^a^	93 (18.5)
**b) Perception towards Cervical Cancer Screening**	Agree	Disagree	Undecided
Cervical cancer screening is a waste of time; it doesn’t stop it from killing someone	6 (1.6)	273(73.4)^a^	93 (25.0)
Cervical cancer screening is only for married women	9 (2.4)	273 (73.4)^a^	90 (24.2)
Pap smear test procedure is too discomforting	50 (13.4)	74 (19.9)^*^	248 (66.7)
The benefits of Cervical cancer screening outweigh the stress of the screening procedure	188 (50.5)^*^	35 (9.4)	149 (40.1)

^a^favourable perception

Most (73.4%) disagreed that CCS is only for married women. 50.5% agreed that the benefits of CCS outweigh the stress of the screening procedure. More details on the respondents’ perceptions are shown in Table 2b below.

### The pattern of utilisation of cervical cancer screening

Only 4% of the respondents have been screened for CC. Their commonly mentioned motivating factor for undergoing the CCS test was the “rate of the increase of CC” (15.89%). Other factors mentioned included “being part of HIV testing” (10.5%), and “done routinely” (10.5%).

Among those that have ever been screened, 46.7% stated that they had been screened in the last 3 years. Pap smear test was the most (53.8%) mentioned type of CCS test received. Only 7.7% of the respondents who had been screened reported that the outcome of the screening test was positiveas shown in Table 3.

**Table 3:** History of involvement in cervical cancer screening test among respondents

History of involvement in cervical cancer screening test	N	%
*Ever been screened for cervical cancer (N* = *372)*		
Yes	15	4.0
No	357	96
*Cervical cancer screening motivating factors (n* = *20)*^a^		
My aunt	1	5
Wedding preparation	1	5
Church advice	1	5
It was free	1	5
Part of HIV testing	2	10
Because it killed Dora Akunyili	1	5
A lot of information in the newspaper	1	5
Done routinely and officially	2	10
Seminar	2	10
Curiosity/to be sure am not infected	1	5
Initiated by the employer in the office	2	10
Rate of increase/ dangers of cervical cancer	3	15
A friend’s advice	1	5
Part of medical checkup	1	5
*Ever been screened for cervical cancer in the last 3 years (n* = *15)*		
Yes	7	46.7
No	8	53.3
*Number of times screened for cervical cancer within the last 3 years (n* = *7)*		
Once	5	71.4
Two times	2	28.6
*Place where last cervical cancer screening test was conducted (n* = *13)*		
Teaching hospitals	3	23.1
Government hospital	1	7.7
A private hospital/clinic	5	38.5
Health programme in school	1	7.7
Workplace/office clinic	2	15.4
Church	1	7.7
*Type of cervical cancer screening test received (n* = *13)*		
Visual inspection	2	15.4
Pap smear test	7	53.8
Human papillomavirus (HPV) testing and pap smear	1	7.7
Don’t know	3	23.1
*Outcome of the cervical cancer screening test (n* = *13)*		
Positive	1	7.7
Negative	12	92.3

^a^multiple responses

### Service delivery preferences relating to the adoption of cervical cancer screening

Most respondents (86.3%) expressed their desire to be screened for CC if given the opportunity. The most preferred place for the screening mentioned was teaching hospitals (60.7%). Some mentioned “anywhere” (2.2%), and any available hospital (0.9%) as their most preferred places. Among the health professionals who preferred to conduct the screening, female doctors 73.2% topped the list followed by any skilled health personnel (20.6%). Some mentioned male doctors (4%) and male nurses (0.3%) as their preferred health professionals as depicted in Table 4.

**Table 4:** Cervical Cancer screening-related intentions and preferences among respondents

**Cervical Cancer screening related intentions and preferences **	**No.**	**Percent (%)**
*Desire to be screened for cervical cancer if given the opportunity (N= 372)*		
Yes	321	86.3
No	51	13.7
*Place where respondents would like to be screened (n= 321)*		
Teaching hospital	195	60.7
University Health centre	33	10.3
Government hospital	29	9.0
A private hospital	44	13.7
Air force hospital	2	0.6
A hospital with experienced personnel/equipment for the test	7	2.2
Any available hospital	3	0.9
Anywhere	7	2.2
Private standard labouratory	1	0.3
*Health professionals preferred to conduct the cervical cancer screening ( n = 321)*		
Female Doctor	235	73.2
Male doctor	13	4.0
Female nurse	6	1.9
Male nurse	1	0.3
Any skilled health personnel	66	20.6
**Preferred cervical cancer screening service delivery options** *N = 372*	**Yes (%)**	**No (%) **
Inclusion of Cervical Cancer Screening test during antenatal visit	263 (70.7)	109 (29.3)
Inclusion of Cervical Cancer Screening test during HIV testing and counseling	225 (60.5)	147 (39.5)
Inclusion of Cervical Cancer Screening test during student entrance medical examination	167 (44.9)	205 (55.1)
Provision of “walk-in” clinics in the hostels where Cervical Cancer Screening test is done	245 (65.9)	127 (34.1)
Rendering Cervical Cancer Screening services in religious centres	239 (64.2)	133 (35.8)

Among the list of preferred CCS service delivery options, most (70.7%) said “inclusion of CCS test during an antenatal clinic visit, 65.9% said “walk-in clinics in the hostel where CCS is done” while 64.2% said they preferred “rendering of CCS services in religious centres/institution”. The least preferred option mentioned was the inclusion of a CCS test during student entrance medical examination as also shown in Table 4.

### Motivation to utilise cervical cancer screening

In Table 5, most respondents (68.8%) stated that they will go for CCS once they have good knowledge of what it entails while 56.5% were willing to use CCS service once the doctor recommends it. More than half of the respondents (56.5%) also disagreed with the statement that they would not go for CCS because they cannot have CC. Slightly more than half of the respondents (53.8%) were undecided about using CCS services no matter the cost while 46.8% were also undecided about obtaining the services without their husbands’ consent.

**Table 5:** Perceived factors that would motivate respondents to utilise Cervical Cancer screening services (*N* = 372)

**Perceived factors that would motivate respondents**	Yes	No	Undecided
I will go for Cervical Cancer Screening once I have good knowledge of what it entails	265 (68.8)	32 (8.6)	84 (22.6)
I will not go for Cervical Cancer Screening because I do not see the need for it	22 (5.9)	225 (60.9)	125(33.6)
I am ready to use Cervical Cancer Screening services no matter the cost	102 (27.4)	70 (18.8)	200 (53.8)
I will go for Cervical Cancer Screening once I see someone who has cervical cancer	18 (4.8)	220 (59.1)	134 (36.0)
I will not go for Cervical Cancer Screening because I cannot have cervical cancer	28 (7.5)	210 (56.5)	134 (36.0)
I am willing to use any Cervical Cancer Screening services once my doctor recommends it	210 (56.5)	38 (10.2)	124(33.3)
I will not go for Cervical Cancer Screening without the consent of my husband	88 (23.7)	110(29.6)	174 (46.8)

### Experiences relating to Sexually Transmitted Infections (STIs), sexual partners, and patterns of condom use

On the question on sexual partnership, 17.8% stated that they currently have one male friend they have sex with while 2.2% said they have more than two male friends. Only 27.7% of the respondents had used condoms, more than half (61.2%) used it rarely while 36.9% used it always. About 55.3% used condoms during the last sexual intercourse as depicted in Table 6.

**Table 6:** Respondents’ exposure to Sexually Transmitted Infections (STIs), Sexual partnership, and patterns of condom use

Exposure to Sexually Transmitted Infections (STIs), Sexual partnership, and patterns of condom use among respondents	No	Percent(%)
*Number of sexual partners currently have sex with (n* = *371)*		
None	297	80
1	66	17.8
More than 1	8	2.2
*Ever used condom(N* = *372)*		
Yes	103	27.7
No	269	72.3
*Type of condom used (n* = *103)*		
Male condom	95	92.2
Female condom	8	7.8
*Frequency of condom use (n* = *103)*		
Always	38	36.9
Rarely	63	61.2
Whenever my partner wants it	2	1.9
*Use of condom during the last sexual activity (n* = *103)*		
Yes	46	44.7
No	57	55.3
*Ever had sexually transmitted infections (N* = *372)*		
Yes	7	1.9
No	365	98.1
Types of sexually transmitted infections ever experienced* (n = *6)*		
Candidiasis	3	50
Staphylococcus	2	33.3
I cannot remember	1	16.7
Age when had sexually transmitted infections* *(n* = *6***)**		
15–19	1	16.7
20–24	4	66.6
25–29	1	16.7

^*^Open responses

^*^Mean age when had STI = 22.2 ± 3.8, Median = 21.5, Mode = 18a, Range = 18–29. a. multiple modes exist. The smallest value is shown

### Association between sexual activity and cervical cancer screening behaviours

Statistical analyses, based on the Pearson square Chi-2 test showed a significant association (*p*.value = 0.0007) between CCS behaviors and sexual activity among women. Results further underscore that the underlying relationship was remarkably high (Yule coefficient = 0.71 > 0.70). Indeed, slightly less than one sexually active woman (ever had sex) in every ten (8.94%) have been screened at least once. Among those who never had sex only 1.60% have been tested at least once. The Odds Ratio of 6.02 shows that sexually active women were six times more likely to develop a pattern of CCS relative to non-sexually active women. Since the 95% confidence interval value of the Odds Ratio does not include the value one, it can be concluded that this value is statistically significant: meaning that it is not likely that the recorded association is due to chance. Table 7 below presents more details on the relationship.

**Table 7:** Relationship between sexual activity and patterns of cervical cancer screening use

	**Has been tested at least once**	**Has never been tested**	**Total**
**Has ever had sex**	11 (8.94%)	112 (91.06%)	123 (100.00%)
**Never had sex**	4 (1.60%)	245 (98.94%)	249 (100.00%)
**Total**	15 (4.00%)	357 (96.00%)	372 (100.00%)
Chi-square Observed = 11.45	Critical Chi-Square = 3.48	P = 0.0007	Q of Yule = 0.71
OR = 6.02		OR-CI (95%) = [2.13; 17.03]	

## Review of the health belief model

The key variables highlighted include the demographic variables, perceived severity of CC, perceived susceptibility to CC, perceived benefits of CCS, perceived barriers to CCS, the likelihood of CCS uptake, cues to action to utilize CCS, and self-efficacy, or confidence in one’s ability to take action.

**Fig. 1:**
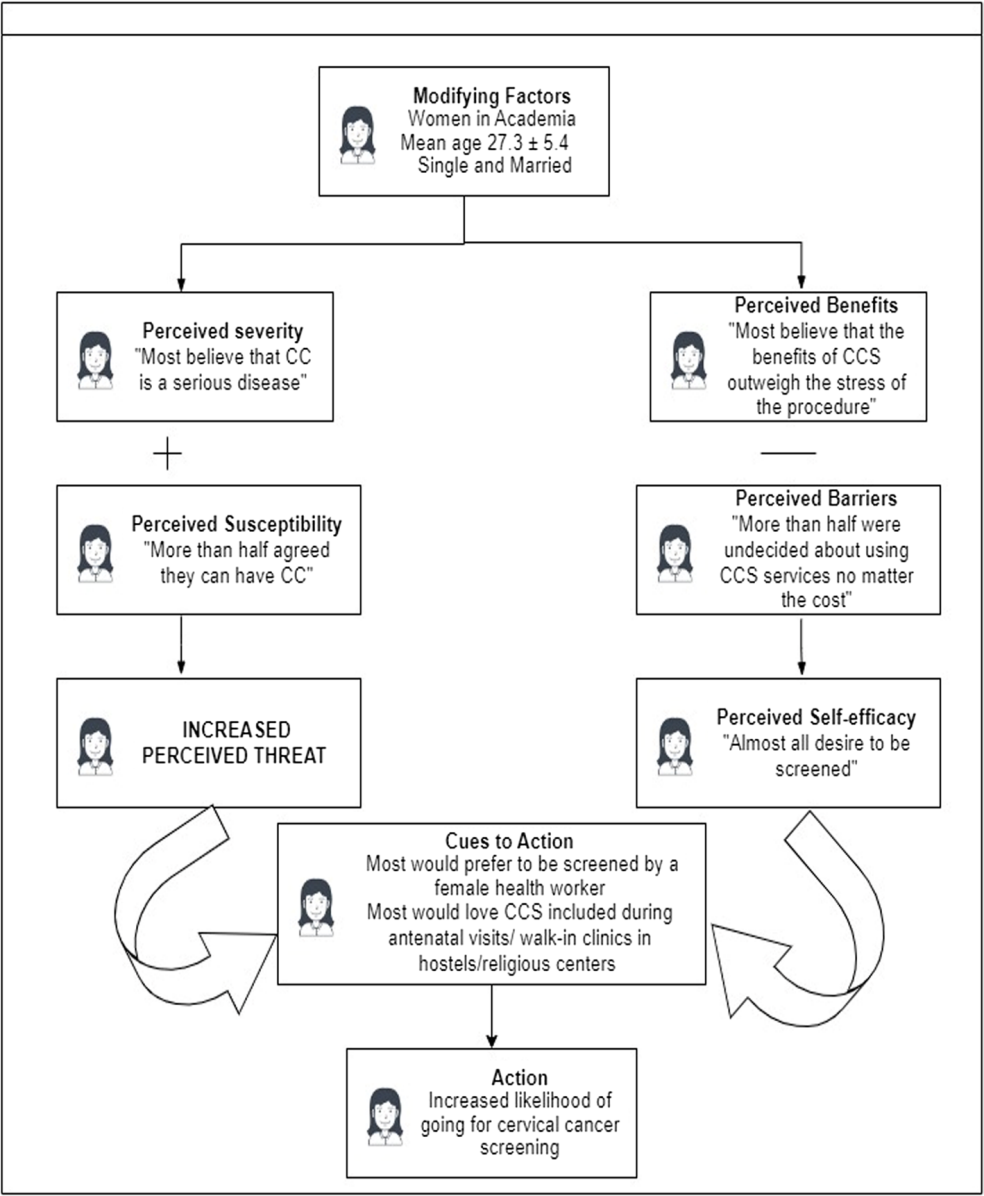
Health belief model applied to study cervical cancer screening adoption

This study indicated that high educational attainment (modifying factors) does not necessarily lead to increased uptake of CCS (Action). Most Nigerian women in this study believe that CC is a serious disease (perceived severity), that they can be exposed to (perceived susceptibility). However, they perceive the cost of CCS and the possibility of the procedure being stressful as part of their limitations (perceived barriers) to assessing CCS. On the other hand, most still believed that the benefit of CCS outweighs the stress of the procedure (perceived benefit), hence high intent to be screened (perceived self-efficacy). Nonetheless, some major factors (Cues to Action) would need to be put in place for an increased likelihood of going for CCS (Action). These factors include CCS being conducted by a female health worker, spousal consent, and CCS being provided during maternity visits, at religious centers, and walk-in clinics within hostels (See Fig. 1).

## Discussion

The study assessed cervical cancer screening behaviors among Nigerian women in academics undergoing a postgraduate study at the time, using the HBM. The results of respondents’ past sexual experience (increased rate of early sexual initiation and poor condom use) showed that there was an extent of exposure among the postgraduate students to some risk factors that can influence the development of CC. It has been noted that delaying the commencement of sexual activity is a strong consideration in the fight against CC [15]. However, only 4% of the respondents have ever been screened for CC. The figure is almost in tune (though lower) with the screening coverage levels in Nigeria and most of sub-Saharan Africa which is generally below 10% [16]. Previous studies had reported values of 8.7% among health workers in South East Nigeria [17] and 15% among market women in Zaria, Northern Nigeria [18].

Of the respondents who have been screened, 7.7% reported a positive screening test result. This is similar to another study [19] which assessed an overall prevalence of cervical abnormalities of 7.6% in Ibadan. It is worrisome to record such a poor uptake rate among the respondents despite the availability of cervical screening services at the University College Hospital. In a similar report from South East Nigeria [17], poor practice and utilization of cervical cancer screening were found among female health workers who are within tertiary facilities where this service is being provided.

There was an optimum desire to be screened for cervical cancer by most of the respondents if given the opportunity, but a greater proportion indicated a preference for female doctors or nurses as they would be more comfortable relating with health workers of the same sex and for privacy. This is as reported in another study where women would prefer not to share critical and complex medical information or be supervised at childbirth by a male doctor [20]. The high preference for female health workers could also be one of the predicting factors influencing a low uptake. Ensuring the availability of female doctors and other health workers incomprehensive maternal healthcare services should be considered an apparent, strategy that might influence the optimum utilization of maternal healthcare services [21]. However, a more recent study reported the poor attitude of female health workers offering the services as one of the factors that negatively affect the uptake of CCS [10], hence the need for training and re-training on service delivery attitudinal change.

Among the list of preferred CCS service delivery options, most suggested the “inclusion of CCS test during antenatal clinic visits.” This was followed by those who recommended the establishment of “walk-in clinics” for conducting CCS services in the hostels, while others preferred “obtaining CCS services in religious centers” as some women are always more confident utilizing health services provided in their religious settings. On the other hand, these places might have been chosen for convenience since they would not need to make extra time to visit the health facility. Certainly, to improve the uptake of cervical screening services among women within Academic institutions in Nigeria, there is a need for a wider qualitative study that will provide the groundwork for effective interventions.

This study showed high motivation and willingness to utilize the screening services. The same level of willingness has been reported by some studies [22, 23]. About half of the respondents were undecided about using CCS services no matter the cost. This might be because of their current academic pursuit which gives them little or no room to source for financial income thereby making them economically under-empowered. Another study among female health workers had previously reported socio-economic status as a strong influence on the uptake of CCS [10]. Almost half of the respondents were also undecided about going for CCS services without their husbands’ consent. This is not surprising considering the patriarchal nature and cultural beliefs of the Nigerian society which gives the men the sole responsibility to make decisions in the family, just as reported in another study where the male study participants insisted that their wives must obtain their consent before screening [24].

As reported in related studies [25–28] this study also showed an association between women’s sexual experiences and health behaviour with their CCS behaviours. The results revealed that sexually active women were six times more likely to develop a pattern of CCS relative to non-sexually active women.

## Conclusion and recommendations

Virtually all the respondents have not been screened for CC and this serves as a pointer to the large extent to which cases of this cancer go without being detected at least till advanced stages have emerged. The optimum desire to be screened by most of the respondents is an opportunity to be maximized bearing in mind their perceived barriers and preferred screening location and health professional for service uptake. Furthermore, despite the high motivation to get screened, the majority were still undecided about accessing the service due to cost and spousal consent. This also reflects that service cost and patriarchal family structure remain limiting factors in the uptake of CCS services, and therefore should be a major consideration in the design of service delivery patterns.

There should be stakeholder collaboration to maximise the high desire to be screened by the respondents to promote the uptake of cervical cancer screening services. This should consider the respondents’ preferred place of screening. Location-specific screening centres can be set up within maternity centres in the school health facility while medical outreaches can be carried out at the University residence, the school youth-friendly centre and at religious centres. Just as reported in the study, cervical cancer screening centres should engage more female health workers who are involved in the screening procedure and the services should be provided at little or no cost.

Self-sampling cervicovaginal secretions for HPV DNA testing has shown to be an effective tool for screening among women. However, proper funding is needed for the transfer of self-collected samples and their analysis. Ensuring that test results are confidential and that proper follow-up care is provided is also crucial.

Though there are major programs focused on CCS and treatment for women which are being implemented, the government and organisations need to roll out and fund more creative and innovative strategies that will explore service delivery options for larger coverage. However, to reduce cervical cancer incidence within the country, it is especially important to design screening programs that will target women who are at high risk for developing CC and as well leverage the existing national immunization system to increase HPV vaccination coverage among young girls aged 9–13 years.

## Study limitations

The research conducted was limited to postgraduate women residing in the University of Ibadan's student residence. As a result, women in academics residing outside the school residence or from a different institution were excluded. Therefore, the findings obtained may not enhance generalization to other populations of women, but they can still provide valuable data for further studies and program interventions.

## Data Availability

Data analyzed are available upon request from the corresponding author.

## Abbreviations

CC: Cervical Cancer
CCS: Cervical Cancer Screening
WHO: World Health Organisation
STI: Sexually Transmitted Infections
HPV: Human Papillomavirus
DNA: Deoxyribonucleic acid
UCH: University College Hospital
HBM: Health Belief Model
